# Supplementary material for: Compensating transport trends in the Drake Passage frontal regions yield no acceleration in net transport
Source: Nat Commun. 2023 Nov 28;14:7792. doi: 10.1038/s41467-023-43499-2 (PMC10684652; doi:10.1038/s41467-023-43499-2)
Supplement: Supplementary file 1 — Supplementary Information [file 41467_2023_43499_MOESM1_ESM.pdf]

Supplementary information for "Compensating transport trends in the Drake Passage frontal regions yield no acceleration in net transport"

Manuel O. Gutierrez-Villanueva[1*], Teresa K. Chereskin[1] and Janet Sprintall[1]

[1*]Scripps Institution of Oceanography, University of California San Diego, 9500 Gilman Dr, La Jolla, 92093, California, USA.

*Corresponding author(s). E-mail(s): mog002@ucsd.edu;
Contributing authors: tchereskin@ucsd.edu; jsprintall@ucsd.edu;

1 Supplementary Text

1.1 Correcting ADCP misalignment angle

Following [1], we analyzed the time series of net total Drake Passage transport $U_{tot} = \int_0^L \int_{z_{max}}^0 u_{tot} dzdx$ (L is the length of the transect), separating the southbound and northbound transects. We vertically integrated the cross-transect total velocity from $z_{max} = -970$ m to the surface to compare our results with those found by [1] for the upper 1042 m. Our z_{max} is shallower than [1] as the cutoff depth, based on 30% good data return, was shallower for the 15-year time series. For the main manuscript, we used $z_{max} = -760$ m as it is the maximum common depth between the XBT/XCTD and cross-transect velocity profiles (see Methods).

As in [1], the total transport calculated from the OS38 velocity shows a systematic offset between the northbound and southbound transport time series. The mean southbound transport was 23 Sv less than that estimated from the northbound cruises. This bias arises from a residual transducer misalignment, unresolved by our bottom-track calibration, that results in a component of the ship's speed erroneously projected in the cross-track direction. We employed

two methods to correct the bias following [1]: the first is based on minimizing transport bias; the second is based on correcting the cross-track velocity for bias. Both methods assume that the true transport is uncorrelated with the direction of travel and that any difference between the mean northbound and southbound transport is due to a residual transducer misalignment resulting in cross-track velocity bias. For the first method, a least-squares model was fitted to the northbound and southbound transport time series:

$$\hat{y} = a_0 + a_1 t + a_2 \cos(\omega_{an} t) + a_3 \sin(\omega_{an} t) + a_4 \cos(\omega_{semi} t) + a_5 \sin(\omega_{semi} t), \quad \text{(S1)}$$

where $\hat{y}$ is the predicted variable (transport), $a_0, ..., a_5$ are the model coefficients corresponding to the mean, trend, annual and semiannual transports, and ω_{an} and ω_{semi} are the annual and semiannual frequencies. U_{tot} estimates are discarded from the least-squares fit if the transport exceeds 3σ, where σ is the standard deviation of the northbound and southbound transects, separately. For the southbound (131) and northbound (117) transects, a_0 was estimated as 75.73 ± 7.06 Sv and 102.56 ± 5.94 Sv, respectively, hence resulting in a bias of 13.41 Sv. The bias was then added to or subtracted from the individual southbound and northbound estimates. Using the least-squares model (eq. S1), the corrected U_{tot} time series yields a mean transport $\overline{U}_{tot} = 89.41 \pm 4.69$ Sv. This value is in close agreement with [1]'s mean of 95 ± 2.1 Sv based on the first 5 years of the same data set, using only those transects falling along the most repeated crossing.

The second method determines a mean velocity offset used to correct the transport calculated for northbound and southbound transects. The velocity offset u_{offs} was estimated as:

$$u_{offs} = \frac{U_{tot} - \overline{U}_{tot}}{A}, \quad \text{(S2)}$$

where A is the area that corresponds to the along-track distance (that varies for each transect) multiplied by the depth of the deepest ADCP bin (970 m). The average u_{offs} for the southbound and northbound transects is 1.4 cm s^{-1} and -1.2 cm s^{-1}, respectively. These values are in agreement with those estimated by [1]. The mean transport using u_{tot} corrected with the velocity offset u_{offs} is not statistically different from that using the transport correction method. The estimated transducer misalignment angle Ω_{miss} is:

$$\Omega_{miss} = \arcsin\left(\frac{u_{offs}}{u_{ship}}\right), \quad \text{(S3)}$$

where $u_{ship} = 5$ m s^{-1} is the typical ship's speed. Excluding a few outliers (5 of 248 transects), the mean Ω_{miss} is $0.14°$ with a standard error of $0.03°$, in agreement with that estimated by [1]. For calculations involving both components of the velocity vector, the additional misalignment angle correction was applied to the 5-min-averaged ADCP and ship velocity time series by

$$\mathbf{u}(t) = \mathbf{u}_{ADCP}(t)e^{-i\Omega_{miss}} + \mathbf{u}_{ship}(t), \tag{S4}$$

where t is the time of the 5-min average, $\mathbf{u} = u + iv$ denotes the horizontal velocity vector in complex notation, and $\mathbf{u}_{ADCP}$ already includes the misalignment angle correction derived from the bottom-track calibration [1]. Equation S4 results in the same average cross-track velocity offsets for northbound and southbound transects as determined by equation S3. Additionally, it provides the correction for the east and north velocity components, as needed for calculating the eddy kinetic energy and eddy momentum flux.

1.2 Calculating wind stress curl

To determine whether the heave-driven cooling found in the Polar Front is consistent with our interpretation of increased Ekman suction in Drake Passage, we estimated time series of wind stress curl $\mathbf{k}\cdot\boldsymbol{\nabla} \times \boldsymbol{\tau}$ for the Southern Ocean (latitude $< 40°$ S), where $\boldsymbol{\tau}$ is the wind stress vector [N m^{-2}], $\mathbf{k}$ is the vertical unit vector, and $\boldsymbol{\nabla}$ is the differential vector operator. We employed daily fields of wind stress from the European Center for Medium-Range Weather Forecast (ECMWF) Reanalysis V5 (ERA-5), which is the latest generation of reanalysis product with a $1/4°$ horizontal resolution [2]; the dataset was downloaded from the Copernicus Climate Change Service (C3S) (2023).

80 # 2 Supplementary Figures

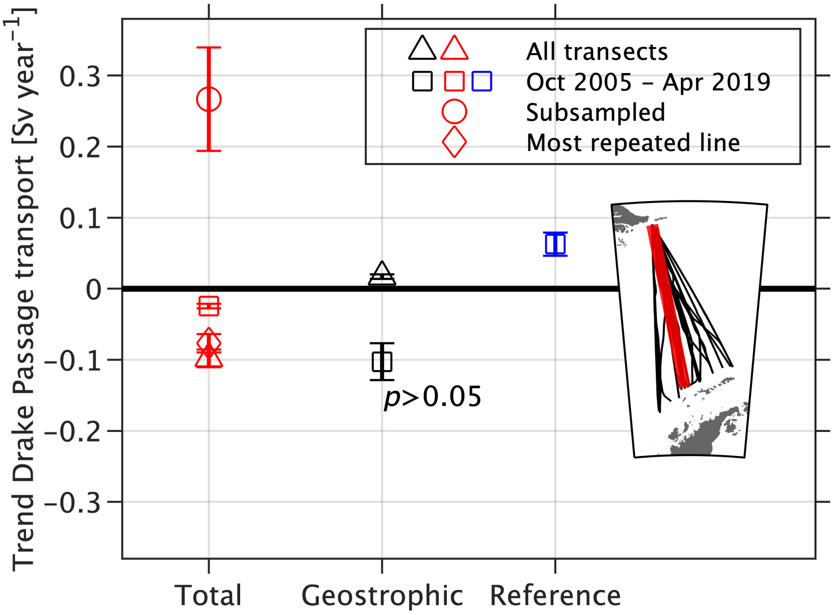

Fig. S1 Trends of net total (red), geostrophic (black), and reference (blue) Drake Passage transport [Sv year^{-1}]. Triangles indicate that all available transects are employed. Trends estimated for the period of October 2005 - April 2019 are marked with squares. Red circle and diamond show the trends estimated with those velocity transects that have a coincident temperature/salinity (XBT/XCTD) transect (subsampled) and along the most repeated line (red thick line in small inset; see Methods), respectively. None of the trends are statistically significant after the modified Mann-Kendall test ($p > 0.05$).

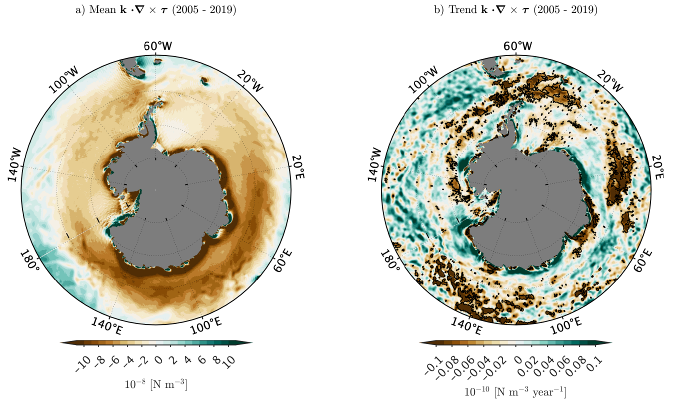

Fig. S2 Wind stress curl over the Southern Ocean for 2005-2019. (a) Time-mean wind stress curl $\mathbf{k} \cdot \nabla \times \boldsymbol{\tau}$ [N m^{-3}]. Negative values indicate Ekman suction (upwelling). (b) Trend in wind stress curl [N m^{-3} year^{-1}] for the last 15 years. Negative values indicate increasing Ekman suction. Trends enclosed with gray solid contour are 95% statistically significant after the modified Mann-Kendall test.

Supplementary References

[1] Firing, Y.L., Chereskin, T.K., Mazloff, M.R.: Vertical structure and transport of the Antarctic Circumpolar Current in Drake Passage from direct velocity observations. J. Geophys. Res. **116**(C8) (2011) https://doi.org/10.1029/2011JC006999

[2] Hersbach, H., Bell, B., Berrisford, P., Biavati, G., Horányi, A., Muñoz Sabater, J., Nicolas, J., Peubey, C., Radu, R., Rozum, I., et al.: ERA5 hourly data on single levels from 1979 to present. Copernicus climate change service (c3s) climate data store (cds) **10**(10.24381) (2018)
